# Combined scleral buckling and phacoemulsification

**DOI:** 10.4103/0974-620X.48416

**Published:** 2009

**Authors:** Pukhraj Rishi, Tarun Sharma, Ekta Rishi, Soumendra P. Chaudhary

**Affiliations:** Shri Bhagwan Mahavir Vitreo Retinal Services, Sankara Nethralaya, Chennai, India

**Keywords:** Cataract, phacoemulsification, retinal detachment, scleral buckling

## Abstract

**Aims:**

To analyze the outcome of combined scleral buckling and phacoemulsification for primary rhegmatogenous retinal detachment and visually significant cataract.

**Settings and Design:**

Retrospective, interventional case series.

**Materials and Methods:**

Retrospective review of patients with primary rhegmatogenous detachment with or without proliferative vitreoretinopathy (PVR) changes and visually significant cataract who underwent combined scleral buckling and phacoemulsification with or without intraocular lens (IOL) implantation between 1991 and 2006. Of the ten eyes, all underwent phacoemulsification and five eyes had posterior chamber Intraocular lens (PC IOL) implantation. All eyes underwent scleral buckling with solid silicone explant combined with #240 encircling band. Subretinal fluid (SRF) drainage was done in five eyes

**Results:**

Four eyes improved to better than 6/12 at a mean follow-up of 9 months. Anatomical success was achieved in all eyes (100%).

**Conclusion:**

Combined scleral buckling and phacoemulsification is a safe and effective procedure that spares the patient the burden of repeated surgeries. It may be considered as a treatment option in selected cases of rhegmatogenous retinal detachment with significant cataract with/without early PVR.

## Introduction

Retinal detachment with cataract presents a unique situation for the vitreoretinal surgeon. In cases where the fundus details cannot be adequately visualized due to cataract, extraction of the lens is performed simultaneously along with the retinal or vitreous surgery. Cataract surgery has undergone vast advancements in recent times and phacoemulsification has become the preferred surgical modality. Small incision wound used in phacoemulsification provides early wound healing and greater structural stability even as the eye contour is well maintained.

Combined phacoemulsification and scleral buckling for rhegmatogenous detachment and cataract was first described by Lazar *et al*.[1] Subsequently, Pierce *et al*. and Girard *et al*., described the triple surgical procedure, phacoemulsification, intraocular lens implantation and scleral buckling for rhegmatogenous retinal detachment with significant cataract.[2,3] They have described successful anatomical and functional outcomes after this procedure.

The purpose of this study is to share our experience, while analyzing the treatment outcomes, with combined phacoemulsification with/without intraocular lens implantation and scleral buckling.

## Materials and Methods

This retrospective study analyzed ten eyes of ten patients who underwent combined phacoemulsification with or without intraocular lens implantation and scleral buckling for rhegmatogenous retinal detachment and dense cataract from September 1991 to September 2006.

All patients had rhegmatogenous retinal detachment without advanced PVR and a significant cataract (NS-III or more with significant PSC cataract). A detailed biomicroscopic examination and fundus evaluation (to the extent possible) was done. Systemic examination was unremarkable in all the patients.

For all patients, preoperative IOL power calculation was carried out while retina was detached, with the other eye serving as a control. In all cases, additional myopia was considered to be approximately -1.00 DS by scleral buckle and -2.00 DS by encircling band. Corrected IOL power was calculated based on the aforesaid considerations. Although we did not measure the post-operative axial length, we believe that it would be a good technique to ‘refine’ the calculation of IOL power, incorporating the ‘surgeon factor’.

All patients underwent surgery under local, peribulbar anesthesia. Phacoemulsification was done through sclero-corneal tunnel placed about 2 mm behind the superior limbus. Anterior chamber (AC) was formed with viscoelastic substance. Limited anterior vitrectomy was done in one case with vitreous loss. A well-dilated pupil and clear media were maintained following the procedure, facilitating intraoperative examination of the fundus details. Retinal breaks were localized with a binocular indirect ophthalmoscope and cryopexy was done. Scleral buckle was then placed and transscleral subretinal fluid drainage was done with a 10/0 suture needle, in five of the ten eyes. Intraocular saline was injected to counter hypotony, wherever necessary. Intravitreal gas was injected in one eye. Lastly, rigid polymethylmethacrylate intraocular lens (5.5 mm optic size) was placed in the capsular bag. Viscoelastic was washed and scleral tunnel sutured with a single, 10-0 nylon mattress suture in all cases. At the end of the procedure, a good external tamponading effect was noted in all eyes.

All patients received topical steroids and cycloplegic agents, postoperatively. Carbonic anhydrase inhibitors were used to control raised intraocular pressure, postoperatively, wherever necessary. All patients were followed up closely for first postoperative week, followed by a review after six weeks. Treatment outcomes were analyzed till most recent follow-up. Postoperative follow-up ranged from two months to three years with one patient being lost to follow-up.

## Results

Ten eyes of 10 patients underwent the combined surgery. All patients were males and the average age was 45.5 years (Range: 17-77 years). The duration from occurrence of detachment to surgical intervention ranged from nine days to one year. Five of ten patients had a traumatic cataract; rest had age-related nucleus sclerosis (grade II to III) and posterior sub-capsular cataract. Pre-operative, best-corrected visual acuity ranged from bare perception of light to 6/36 (Snellen`s).

Seven of the ten patients had retinal retinal detachment extending more than two quadrants and the macula was detached in five cases (50%). Three patients had traumatic retinal dialysis and the rest had breaks related to posterior vitreous detachment. Only one patient had lattice degeneration with holes associated with high myopia. One patient each, had associated (mild) vitreous hemorrhage, non proliferative diabetic retinopathy and primary open angle glaucoma. Two patients had post traumatic angle recession. Three eyes each had grade A and grade B PVR changes [Table 1].

**Table 1:** Clinical features of retinal detachment at presentation

*Features of the retinal*	*N*	*Percentage*
*detachment at presentation*
>2 quadrants	7	70
Macula off	5	50
No PVR	4	40
PVR grade A	3	30
PVR grade B	3	30
PVR grade C	0	0
Dialysis (Traumatic)	3	30
Flap tears	6	60
Atrophic hole and Lattice	1	10
No breaks seen	0	0

All patients underwent phacoemulsification; one patient had posterior capsular rupture with vitreous loss for which limited anterior vitrectomy was done. During the retinal surgery, no difficulty related to cataract wound *viz* anterior chamber collapse or iris prolapse was encountered.

In all cases, a solid silicone explant combined with #240 encircling band was used. Sub-retinal fluid was drained in five eyes. One patient had localized choroidal hemorrhage and one patient had vitreous incarceration at the site of SRF drainage (20%). Rest of the patients did not have any surgery-related complications. In one patient, 0.3cc of 100% C3F8 was used as an endotamponade.

After the retinal procedure, five eyes underwent posterior chamber IOL implantation. The five eyes in which IOL implantation was not possible was because of change in surgical plan, per-operatively to switch from vitreous surgery alone to scleral buckling and cataract surgery. All these patients were given the option of a secondary IOL implantation versus contact lens use. Four of these patients continued contact lens wear while one was lost to follow-up. We did not encounter any intraocular lens subluxation or dislocation. In one patient with preexisting primary open angle glaucoma, trabeculectomy (with Kelly`s punch) was done after phacoemulsification and scleral buckling, in the same sitting. Nine of ten patients achieved retinal re-attachment, six weeks postoperatively. One patient with retinal reattachment at two weeks of post-operative period was subsequently lost to follow-up. Four patients required medication for postoperative high intraocular pressure that also included the patient with preoperative secondary glaucoma (40%).

Visual acuity improved in eight cases while one patient maintained the preoperative vision. Four patients attained best corrected visual acuity (BCVA) more than 6/12, N10 and one patient was lost to follow-up [Table 2]. All patients with attached macula had good visual recovery.

**Table 2:** Pre and post-operative details

*Case*	*Age (Yrs)*	*Sex*	*Macula (Pre-op)*	*Preoperative VA*	*Anatomical*	*Post-operative*	*Post - op*	*Follow-up*
					*outcome*	*refractive error*	*BCVA*	*(months)*
1	38	M	Attached	6/36, N36	Attached	+10.00 DS	6/9, N6	19
2	65	M	Detached	FCCF, N60	Attached	-2.00 DS/-0.75 DCX90	6/60, N36	3
3	21	M	Detached	FCCF, N36	Attached	-0.75 DS/-0.75 DCX120	3/60, N24	2
4	43	M	Attached	2/60, N36	Attached	-2.50 DCX110	3/60, N24	2
5	42	M	Attached	FCCF, N36	Attached	-1.00 DS/-0.50 DCX160	6/12, N10	12
6	64	M	Attached	FCCF, N36	Attached	+ 9.50 DS	6/6, N8	42
7	62	M	Detached	FCCF, N36	Attached	-	-	Lost to follow-up
8	26	M	Attached	6/60, N36	Attached	+ 9.75 DS	6/6, N6	2
9	77	M	Detached	2/60, N36	Attached	+ 11.00 DS	3/60, N36	24
10	17	M	Detached, Scar	FCCF, N36	Attached	- 3.50 DS	FCCF, N36	2

## Discussion

The combination of retinal detachment and dense cataract presents a unique situation to the vitreoretinal surgeon. It is difficult to manage with conventional techniques due to instability of the (extracapsular cataract surgery) wound and difficulty in maintaining the eyeball contour. Combined cataract and retinal surgery has been employed to increase anatomical and functional outcomes. Amongst the available options of retinal reattachment surgery, the single operation success of scleral buckling makes it an attractive option in a (particularly) given surgical situation.[4,5] A large majority of scleral buckling cases do not require an endotamponade, thereby not interfering with the stability of the IOL. Nor is a second surgery required for removal of the endotamponade, making it a less morbid procedure for the eye. In such instances, combining phacoemulsification with scleral buckling rather than vitrectomy may be a more optimal surgical decision. However, proper case selection (fresh rhegmatogenous retinal detachment with minimal PVR) and surgeon’s familiarity with the surgical techniques must be factored into achieving higher success rates. Conventional large incision cataract surgeries cannot be combined with scleral buckling because of the instability of the wound and inability to maintain the eyeball contour because of fluctuation in intraocular pressure.[6] Also, there is an increased risk of vitreous loss and aggravation of retinal condition. Phacomulsification provides a better stability of the wound during the procedure because of its small size.

In 1977, Lazar *et al*., first reported a case of combined phacoemulsification and scleral buckling procedure in a 30-year-old mentally retarded female with a successful anatomical outcome.[1] Pierce *et al*., initially reported simultaneous phacoemulsification with posterior chamber IOL implantation and scleral buckling.[2] After that a few studies have shown both successful anatomical and functional outcomes with some modification in the procedure.[3,7]

Girard *et al*. reported a case series of 15 patients who underwent a triple procedure: phacoemulsification, IOL implantation, and scleral buckling for fresh as well as recurrent rhegmatogenous retinal detachment with a significant cataract.[3] They noted high postoperative IOP in only one of their cases, which was attributed to the use of viscoelastic. Overall anatomical success rate was 87% and functional success (visual acuity better than 20/40) was 54%. They concluded that combined phacoemulsification and scleral buckling was a safe and effective procedure. Tsai *et al*. reported combined phacoemulsification with posterior chamber IOL implantation and scleral buckling with anatomical success of 100%, however, sample size (six cases) was small. They did not encounter any case of postoperative high intraocular pressure (IOP).[7]

In our series, 50% of cases had trauma and 50% had long standing retinal detachment. We localized the breaks in the aphakic (post-phacoemulsification) state and implanted the IOL after the scleral buckling, a method that was also followed by Tsai *et al*.[7] Whereas Girard *et al*. performed the scleral buckling after IOL implantation, placing the IOL before the scleral buckling might hamper peripheral visualization and also affect the final outcome. We used rigid polymethylmethacrylate intraocular lens (5.5 mm) as used by Girard *et al*. while Tsai *et al*. used foldable intraocular lens.

In one case, we also performed filtering surgery for pre-existing primary open angle glaucoma. However, the postoperative IOP was high which required antiglaucoma medication again. Viscoelastic was washed at the end of surgery in all cases as leaving viscoelastics increases the risk of high postoperative IOP.[8] In spite of this, three of our cases had postoperative high IOP, which was controlled with topical antiglaucoma medication. One of these patients had pre-operative angle recession, which might have contributed to post operative high IOP. Scleral buckling itself has been reported to raise the IOP to as high as 36 mmHg.[9] We also analyzed the postoperative refractive error, especially in the patients who underwent IOL implantation to check the accuracy of the criteria we employed for IOL power calculation. It ranged between 0 and -3.5 D spherical equivalent; patients had a good acceptance with no complaints of diplopia.

Overall anatomic success rate in our series was 100%, which is similar to that reported in previous studies.[7] Visual acuity improved in 8 cases. Four patients attained BCVA of more than 6/12, N10 while 1 patient maintained the preoperative visual acuity due to macular scar and one patient was lost to follow-up. It has been reported that visual recovery after retinal reattachment is most dependent on pre-operative macular involvement.[10] In our series, poor visual outcome in macula detached cases was considered to be photoreceptor damage due to long-standing retinal detachment.

Combined phacoemulsification and scleral buckling is a safe and effective procedure, which improves the anatomical and functional outcome in cases with coexisting retinal detachment and significant cataract. However, visual outcome depends on the duration of detachment and associated ocular conditions. It spares the patient of undergoing repeated surgeries and also decreases the risk of recurrent detachment. It may be considered as a surgical alternative in selected cases with significant cataract and rhegmatogenous retinal detachments with/without minimal PVR.
